# Supplementary material for: Pyruvate Dehydrogenase Kinase 1 inhibition mediated oxidative phosphorylation enhancement in cartilage promotes osteoarthritis progression
Source: BMC Musculoskelet Disord. 2023 Jul 20;24:597. doi: 10.1186/s12891-023-06585-6 (PMC10357736; doi:10.1186/s12891-023-06585-6)
Supplement: Supplementary file 1 — Additional file 1. [file 12891_2023_6585_MOESM1_ESM.zip › MF.Gsea.1653627316242/GOMF_IMMUNOGLOBULIN_BINDING.html]

Details for gene set GOMF\_IMMUNOGLOBULIN\_BINDING[GSEA]

|  || Dataset | 55457\_collapsed\_to\_symbols.cls.cls #OA\_versus\_normal.cls.cls #OA\_versus\_normal\_repos |
| Phenotype | cls.cls#OA\_versus\_normal\_repos |
| Upregulated in class | OA |
| GeneSet | GOMF\_IMMUNOGLOBULIN\_BINDING |
| Enrichment Score (ES) | 0.37775487 |
| Normalized Enrichment Score (NES) | 1.2397487 |
| Nominal p-value | 0.18639798 |
| FDR q-value | 0.4780098 |
| FWER p-Value | 1.0 |
Table: GSEA Results Summary

  

Fig 1: Enrichment plot: GOMF\_IMMUNOGLOBULIN\_BINDING      
 Profile of the Running ES Score & Positions of GeneSet Members on the Rank Ordered List

  

| SYMBOL | TITLE | RANK IN GENE LIST | RANK METRIC SCORE | RUNNING ES | CORE ENRICHMENT || 1 | FCGR1A | Fc fragment of IgG receptor Ia [Source:HGNC Symbol;Acc:HGNC:3613] | 633 | 0.610 | 0.0384 | Yes |
| 2 | FCGRT | Fc fragment of IgG receptor and transporter [Source:HGNC Symbol;Acc:HGNC:3621] | 800 | 0.570 | 0.1073 | Yes |
| 3 | MS4A2 | membrane spanning 4-domains A2 [Source:HGNC Symbol;Acc:HGNC:7316] | 1203 | 0.475 | 0.1442 | Yes |
| 4 | FCGR2C | Fc fragment of IgG receptor IIc (gene/pseudogene) [Source:HGNC Symbol;Acc:HGNC:15626] | 1460 | 0.435 | 0.1867 | Yes |
| 5 | MS4A1 | membrane spanning 4-domains A1 [Source:HGNC Symbol;Acc:HGNC:7315] | 1652 | 0.406 | 0.2301 | Yes |
| 6 | JCHAIN | joining chain of multimeric IgA and IgM [Source:HGNC Symbol;Acc:HGNC:5713] | 1805 | 0.384 | 0.2734 | Yes |
| 7 | FCGR2B | Fc fragment of IgG receptor IIb [Source:HGNC Symbol;Acc:HGNC:3618] | 2016 | 0.360 | 0.3087 | Yes |
| 8 | LILRA2 | leukocyte immunoglobulin like receptor A2 [Source:HGNC Symbol;Acc:HGNC:6603] | 2444 | 0.309 | 0.3198 | Yes |
| 9 | FCER1A | Fc fragment of IgE receptor Ia [Source:HGNC Symbol;Acc:HGNC:3609] | 2564 | 0.295 | 0.3529 | Yes |
| 10 | FCER1G | Fc fragment of IgE receptor Ig [Source:HGNC Symbol;Acc:HGNC:3611] | 2752 | 0.275 | 0.3778 | Yes |
| 11 | FCER2 | Fc fragment of IgE receptor II [Source:HGNC Symbol;Acc:HGNC:3612] | 3785 | 0.178 | 0.3232 | No |
| 12 | CD22 | CD22 molecule [Source:HGNC Symbol;Acc:HGNC:1643] | 4296 | 0.128 | 0.3020 | No |
| 13 | FCGR3A | Fc fragment of IgG receptor IIIa [Source:HGNC Symbol;Acc:HGNC:3619] | 4594 | 0.105 | 0.2940 | No |
| 14 | PIP | prolactin induced protein [Source:HGNC Symbol;Acc:HGNC:8993] | 5814 | 0.008 | 0.2005 | No |
| 15 | FCGR2A | Fc fragment of IgG receptor IIa [Source:HGNC Symbol;Acc:HGNC:3616] | 6329 | -0.031 | 0.1650 | No |
| 16 | FCAR | Fc fragment of IgA receptor [Source:HGNC Symbol;Acc:HGNC:3608] | 8670 | -0.206 | 0.0128 | No |
| 17 | VWF | von Willebrand factor [Source:HGNC Symbol;Acc:HGNC:12726] | 9180 | -0.246 | 0.0086 | No |
| 18 | FCGR3B | Fc fragment of IgG receptor IIIb [Source:HGNC Symbol;Acc:HGNC:3620] | 9341 | -0.259 | 0.0333 | No |
| 19 | AMBP | alpha-1-microglobulin/bikunin precursor [Source:HGNC Symbol;Acc:HGNC:453] | 9483 | -0.273 | 0.0614 | No |
| 20 | LGALS3 | galectin 3 [Source:HGNC Symbol;Acc:HGNC:6563] | 9508 | -0.274 | 0.0989 | No |
| 21 | HRG | histidine rich glycoprotein [Source:HGNC Symbol;Acc:HGNC:5181] | 9986 | -0.320 | 0.1078 | No |
| 22 | UMOD | uromodulin [Source:HGNC Symbol;Acc:HGNC:12559] | 10605 | -0.384 | 0.1149 | No |
| 23 | DLG4 | discs large MAGUK scaffold protein 4 [Source:HGNC Symbol;Acc:HGNC:2903] | 11077 | -0.441 | 0.1416 | No |
Table: GSEA details [plain text format]

  

Fig 2: GOMF\_IMMUNOGLOBULIN\_BINDING      
 Blue-Pink O' Gram in the Space of the Analyzed GeneSet

  

Fig 3: GOMF\_IMMUNOGLOBULIN\_BINDING: Random ES distribution      
 Gene set null distribution of ES for **GOMF\_IMMUNOGLOBULIN\_BINDING**
